# Evaluation of the routine implementation of pulse oximeters into integrated management of childhood illness (IMCI) guidelines at primary health care level in West Africa: the AIRE mixed-methods research protocol

**DOI:** 10.1186/s12913-022-08982-4

**Published:** 2022-12-24

**Authors:** Gildas Boris Hedible, Sarah Louart, Désiré Neboua, Laura Catala, Gildas Anago, Abdoul-Guaniyi Sawadogo, G. Désiré Kargougou, Bertrand Meda, Jacques Séraphin Kolié, Adama Hema, Sory Keita, Mactar Niome, Abdoul Salam Savadogo, Lucie Peters-Bokol, Honorat Agbeci, Zineb Zair, Severin Lenaud, Marine Vignon, Solange Ouedraogo Yugbare, Hannatou Abarry, Abdoul Aziz Diakite, Ibrahima Sory Diallo, Franck Lamontagne, Valérie Briand, Désiré Lucien Dahourou, Anthony Cousien, Valéry Ridde, Valériane Leroy, for the AIRE Research Study Group

**Affiliations:** 1 Inserm, University Paul Sabatier Toulouse 3, CERPOP, UMR 1295, Toulouse, France; 2 ALIMA, Dakar, Senegal; 3 IRD, CEPED, Paris, France; 4 University of Lille, CLERSE - Centre Lillois d’Études et de Recherches Sociologiques et Économiques, Lille, France; 5 Terre des hommes-Lausanne (Tdh), Ouagadougou, Burkina Faso; 6 ALIMA, Bamako, Mali; 7 SOLTHIS, Niamey, Niger; 8 ALIMA, Conakry, Guinea; 9 Program PACCI, Abidjan, Côte d’Ivoire; 10 CHU de Bogodogo, Ouagadougou, Burkina Faso; 11 Ministère de la santé, des populations et des affaires sociales, Niamey, Niger; 12 CHU Gabriel Touré, Bamako, Mali; 13 Institut de Nutrition et Santé de l’Enfant (INSE), Conakry, Guinea; 14 SOLTHIS, Paris, France; 15 University of Bordeaux, Inserm UMR 1219, IRD EMR 271, Bordeaux Population Health Centre, Bordeaux, France; 16 Institut de Recherche en Sciences de la Santé/CNRST, Département Biomédical, Santé Publique, Ouagadougou, Burkina Faso; 17 Université Paris Cité and Université Sorbonne Paris Nord, Inserm, IAME, F-75018 Paris, France; 18 Center for Epidemiology and Research in Population Health (CERPOP), UMR 1295, Inserm, University Paul Sabatier Toulouse 3, Toulouse, France

**Keywords:** Children, Integrated management of childhood illness, Pulse oximeter, Primary health care, West Africa, Implementation research

## Abstract

**Background:**

The AIRE operational project will evaluate the implementation of the routine Pulse Oximeter (PO) use in the integrated management of childhood illness (IMCI) strategy for children under-5 in primary health care centers (PHC) in West Africa. The introduction of PO should promote the accurate identification of hypoxemia (pulse blood oxygen saturation Sp02 < 90%) among all severe IMCI cases (respiratory and non-respiratory) to prompt their effective case management (oxygen, antibiotics and other required treatments) at hospital. We seek to understand how the routine use of PO integrated in IMCI outpatients works (or not), for whom, in what contexts and with what outcomes.

**Methods:**

The AIRE project is being implemented from 03/2020 to 12/2022 in 202 PHCs in four West African countries (Burkina Faso, Guinea, Mali, Niger) including 16 research PHCs (four per country). The research protocol will assess three complementary components using mixed quantitative and qualitative methods: a) context based on repeated cross-sectional surveys: baseline and aggregated monthly data from all PHCs on infrastructure, staffing, accessibility, equipment, PO use, severe cases and care; b) the process across PHCs by assessing acceptability, fidelity, implementation challenges and realistic evaluation, and c) individual outcomes in the research PHCs: all children under-5 attending IMCI clinics, eligible for PO use will be included with parental consent in a cross-sectional study. Among them, severe IMCI cases will be followed in a prospective cohort to assess their health status at 14 days. We will analyze pathways, patterns of care, and costs of care.

**Discussion:**

This research will identify challenges to the systematic implementation of PO in IMCI consultations, such as health workers practices, frequent turnover, quality of care, etc. Further research will be needed to fully address key questions such as the best time to introduce PO into the IMCI process, the best SpO2 threshold for deciding on hospital referral, and assessing the cost-effectiveness of PO use. The AIRE research will provide health policy makers in West Africa with sufficient evidence on the context, process and outcomes of using PO integrated into IMCI to promote scale-up in all PHCs.

**Trial registration:**

Trial registration number: PACTR202206525204526 retrospectively registered on 06/15/2022.

**Supplementary Information:**

The online version contains supplementary material available at 10.1186/s12913-022-08982-4.

## Background

Despite advances in child health management globally, sub-Saharan Africa continues to be the region with the highest under-5 mortality rate in the world [1]. An estimated 2.7 million children died before their fifth birthday in sub-Saharan Africa in 2017, 61.5% from infectious causes [2]. The leading causes of death globally remain pneumonia (24%), diarrhea (15%) and malaria (9%), with malnutrition being a significant underlying factor linked to around half of all deaths among children aged 1 to 59 months [1]. The highest under-5 mortality are observed in West Africa with a high burden of infectious diseases. There, under-5 mortality is due to neonatal conditions (23.7%), followed by lower respiratory tract infections (16.3%), malaria (14.8%) and diarrhea (13.0%) [3].

### Prevalence and severity of hypoxemia in children

Hypoxemia is defined as hemoglobin oxygen saturation below 90% at sea level, as measured by pulse oximeter (PO), pulse oxygen saturation (SpO2), or blood gas tests (arterial oxygen saturation, SaO2) [4]. Hypoxemia is a common sign of severity in acute respiratory and non-respiratory illness and greatly increases the risk of death [5–7]. According to setting and the severity of disease, it is estimated that at least 2% [8] to 80% [6, 9, 10] of children with serious illnesses including pneumonia have hypoxemia. Outpatient children and those with a clinical diagnosis of acute upper respiratory tract infection were at low risk of hypoxemia (pooled estimate 6 to 9%) [11]. Prevalence increased to 31 and 43% in emergency department patients and clinical pneumonia cases, respectively, and was even higher in hospitalized children (47%) and those with confirmed pneumonia by X-ray (72%). Hypoxemia also occurs in children with serious illness, such as sepsis, meningitis, neonatal infections, anemia, … [8].

In 12 secondary level hospitals in Nigeria, a prospective cohort study was setup among neonates (0–28 days of life) and children (1 month-14 years old) to report the prevalence of hypoxemia, the oxygen utilization and clinical predictors of hypoxemia measured at the hospital entrance [6]. Among the 23,926 participants enrolled, the prevalence of hypoxemia was 22.2% (95% CI: 21.2–23.2) in neonates, and 10.2% (95% CI: 9.7–10.8) in children. It was common in children with acute lower respiratory tract infection (28%), asthma (20%), meningitis (17%), malnutrition (16%), acute febrile encephalopathy (15%), sepsis (9%) and malaria (9%) and in neonates with neonatal encephalopathy (33%), prematurity (27%) and sepsis (21%). In this study, hypoxemia increased the adjusted risk of death by 6-fold in neonates and 7-fold in children [6].

These studies also consistently highlight the fact that hypoxemia is poorly predicted by clinical signs and that their predictive value varied by age and respiratory and non-respiratory conditions. Many studies at the hospital level have explored the usefulness of clinical signs, such as tachypnea, quiet stridor or chest indrawing, cyanosis to identify hypoxemia in children with respiratory disease [12–16], but little has attempted to explore this question in non-respiratory illness. An hospital-based study conducted in Nigeria concludes that individual or combined clinical signs can predict hypoxemia in children with respiratory and non-respiratory illness [6]. But, it is crucial to note that children with serious illness (including malaria, pneumonia, malnutrition or other illnesses) are clinically undiagnosed. Therefore, at primary care level where PO was usually not available, children with hypoxemia (SpO2 < 90%) are often undiagnosed and not timely referred to hospital to be adequately managed (oxygen therapy, artemisinin-based combined therapy [ACT], antibiotics…) [17–19].

This poor clinical identification of severe cases with life-threatening hypoxemia at the PHC level coupled with underqualified and poorly trained health care workers contributes to high levels of child mortality in low- and middle-income countries (LMIC), including West Africa [20].

### Identifying more accurately hypoxemic among severe cases by introducing pulse oximetry at PHC level in LMIC

The pulse oximeter is a simple, non-invasive, reproductible, accurate tool that could help to reduce childhood morbidity and mortality by accurately diagnosing and monitoring children with hypoxemia [20–22]. PO remains the most reliable diagnostic tool of hypoxemia in children that can be used at all levels of the healthcare system in LMICs [19, 23]. While PO have been used at the hospital level in Africa [24–27], their integration at the PHC level has not yet been adopted [28]. Their use, when available, is recommended only for children with cough and/or difficult breathing in the 2014 World Health Organisation (WHO) IMCI guidelines [29, 30], and in the WHO guidelines on oxygen therapy for children [4]. Although there are durability challenges related to battery life, PHC have used the same device for longer than 4 years, demonstrating its robustness in these settings [31]. A modelling study showed that PO could be cost-effective and promising in LMIC to increase the diagnosis of severe cases of hypoxemia and improve their management with oxygen therapy and specific treatments, as well as to reduce the incidence of incorrect treatment with antibiotics [9].

However, few studies have evaluated the field implementation of PO at the PHC level in the LMICs. These studies described the opportunities and barriers to overcome to scale up the use of PO devices to identify and treat hypoxemia early in the PHC.PO was found to be feasible with a median performance time for PO of 42 seconds (interquartile range 37–50), ≤ 1 minute for 94.4% of the children, without adverse events and acceptable by parents/guardians in Pakistan [32]. In Nigeria and in Uganda, a study showed that the use of PO is simple and life-saving, but introducing it into routine practice is also challenging [27] with access to oxygen compromised by faulty equipment, lack of PO and inadequate care practices [25]. Another study in Kenya also highlighted the need for a clear supply and repair circuit and periodic training of health care workers (HCW) to support the PO use [26]. In Malawi [19, 23], the PO use among children with IMCI danger signs or chest indrawing shows that those whose SpO2 was ≤90% were more than twice as likely to be referred at hospital than those with SpO2 > 90%: 84% vs 42% (*p* < 0.001). The diagnosis of hypoxemia using PO has also increased the effective referral rate in asymptomatic children from 0 to 27% (p < 0.001) [19]. Their experience highlighted that PO screening should be routine in outpatient clinics and performed as early as possible in the patient’s care pathway. Training on PO use increased the number of hospital referrals of children with severe hypoxemia [18]. An Ethiopian trial reported that the proportion of children with severe pneumonia reached 16% in the intervention arm using PO versus 3.9% in the control arm without PO, highlighting that the combined use of IMCI and PO at the PHC level increased the number of severe pneumonia cases diagnosed in children [33]. In Uganda, the prevalence of severe hypoxemia using PO (SpO2 ≤ 90%) at PHC level was estimated at 1.3% in children under-5 [17].

Until early 2020, the PO has never been used during the IMCI consultation at PHC level in West Africa. In addition, there remains limited data about the epidemiology, identification, care pathways, care patterns, costs of care and outcomes of children with serious illness at the primary health care level in this context. This also evolved with the emergence of the COVID-19 pandemic in 2020 and required further investigation.

Therefore, we hypothesized that the routine use of PO integrated into IMCI consultations in children < 5 years of age at PHC level will allow for a timely detection of hypoxemia in all respiratory and non-respiratory cases to prompt their effective referral to hospital for an appropriate case management (oxygen, antibiotic therapy and other treatments) in West Africa. The AIRE project is aimed to address: a) the lacks of tool to detect hypoxemia, adapted for routine use at the PHC in West Africa and b) the limited knowledge on how best to operationalize and optimize PO-use within the IMCI approach (current Standard of Care).

### The AIRE project: improved identification of child respiratory distress from the French acronym “Amélioration de l’Identification des Détresses Respiratoires chez l’Enfant”)

From July 2019 to April 2023, the AIRE project, funded by UNITAID is implemented in four West African countries under the auspices of Ministries of Health, by the consortium led by Alliance for International Medical Action (ALIMA), in partnership with Solidarité Thérapeutique et Initiatives pour la Santé (Solthis), Terre des hommes-Lausanne (Tdh), and the National Institute of Health and Medical Research (Inserm).

The AIRE project proposed the implementation of a complex public health intervention with three components: a) equip the intervention districts, introduce the routine use PO integrated into IMCI consultations and increase the community awareness of timely care seeking for sick children, b) generate research evidence (context, process, and outcomes) on the implementation of the use of PO at the decentralized level and c) scale up the use of the PO. The evaluation of the project will be carried out under the field conditions of care, according to the national guidelines in force, and without replacing the health system. The research protocol was developed by Inserm in partnership with the French National Research Institute for Sustainable Development (IRD).

### The AIRE operational intervention strategy

The project implements the routine use of PO device integrated into IMCI consultations by HCW in 202 PHC and in eight District Hospital (DH) in four West African countries (Burkina Faso, Guinea, Mali, Niger). It will help improve the IMCI strategy by using PO alongside targeted community awareness to better identify danger signs and timely referral to PHC of all severe respiratory and non-respiratory cases, while District Hospitals will be equipped with the appropriated treatment (e.g., injectable antibiotics, oxygen therapy).

**Fig. 1:**
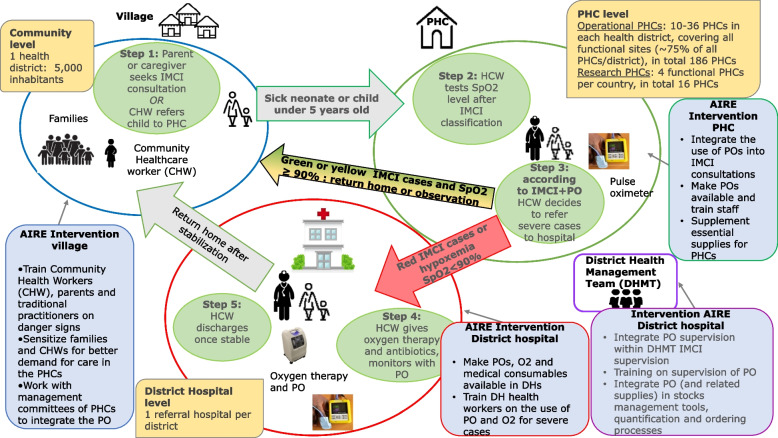
Pathway of sick children through the AIRE operational intervention strategy implemented at the 202 PHC and 8 DH in Burkina Faso, Guinea, Mali, Niger. March 2021–December 2022

From March 2021 to December 2022, the AIRE intervention strategy will propose a care pathway for sick children in five stages (Fig. 1) starting from their home or community level to the referral hospital for severe cases via the PHC and then returning to the patient’s home or community.

The different steps of the AIRE strategy implemented are as follows:

- Step 1, at the community level, the parent/guardian will attend IMCI consultations or community healthcare workers (CHW) will refer sick children to PHC via community outreach.
- Step 2: at the PHC level, PO integration into the routine of care IMCI guidelines

PO will be introduced in all AIRE PHC within a two-month rollout period. This deployment will also include a specific COVID-19 intervention (training of HCW and individual protective equipment for prevention…). AIRE implementing NGOs will deploy at least one PO per PHC (PO AH-MX, manufactured by Acare©). Back-ups will be available at the project staff level and will allow for replacement in the event of faulty purchase orders. A biomedical technician in each country will be in charge of maintenance.

All HCW from PHC and DH will receive a 4–7-day training (except 15 days for Mali) on how to use and maintain the PO embedded in IMCI guidelines. To this end, training materials will be developed by each country in close collaboration with the IMCI technical group using existing IMCI training materials and PO training materials. A pre-test and post-test evaluation will be conducted to assess the knowledge and practice of PO introduction into IMCI. After the training, HCW will receive on-site support during the first weeks of PO implementation from clinical supervisors. In addition to this, regular supervision will be carried out by the project teams, the health district teams, and the national or regional supervisors in order to monitor the effective use of PO, staff turnover (new recruitments, illness, etc.), new health professionals requiring training, the technical difficulties (e.g. broken to be replaced, probes and other accessories to be provided…).

The WHO generic guidelines for IMCI were provided in 2005, then revised in 2014 specifically for pneumonia [29, 30, 34] and were revised for neonates in 2019 [35]. At each PHC, the AIRE project will be conducted with their current national guidelines that present few specificities according to the adaptation of the WHO guidelines. In addition, there will be also a distinction between the countries using paper IMCI support (Niger, Guinea and Mali - Dioila health district) and those using electronic IMCI (e-IMCI). Indeed, Terre des hommes (Tdh) NGO has developed the Integrated electronic Diagnosis Approach (IeDA) [36] in Burkina Faso and Mali - Markala health district. It is a digitalized IMCI algorithm via Commcare software used by HCW to perform IMCI consultations on an electronic consultation register via a tablet.

**Fig. 2:**
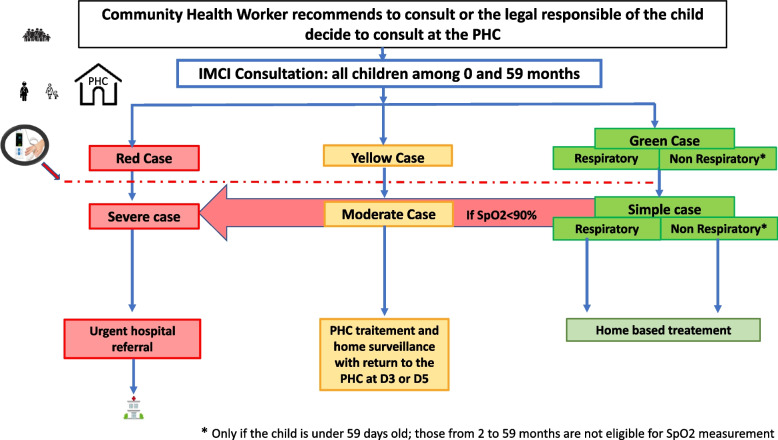
PO integration into IMCI guidelines and classification of IMCI cases in all AIRE sites. Burkina Faso, Guinea, Mali, Niger, March 2021–December 2022

In all AIRE PHCs, HCW will diagnose and treat the child according to paper or electronic-based IMCI guidelines and incorporate the operational use of PO only after the child’s examination and IMCI classification (Fig. 2), given the International Advisory Group recommendation (composed by WHO, Unitaid, Unicef, and expert representatives). The PO will be used among the following eligible children according to the IMCI classification and according to age and signs:

- 0–59 days: all sick children
- 2–59 months, with cough and difficulty breathing: all sick children (green, yellow or red cases classified by IMCI)
- 2–59 months, without cough or difficulty breathing: those classified as yellow or red.

Severe cases defining the “red case” are those with any of the specific danger signs or symptoms of the disease according to the age category defined by the IMCI guidelines, from 0 to 59 days [35] or 2 to 59 months [29, 30]. Children who have not yet been identified as severe cases using only IMCI guidelines (initially classified as green and yellow cases) could be diagnosed as severe cases when using PO, if SpO2 < 90%, defining hypoxemia (red arrow in Fig. 2).

- Step 3: Decision on Hospital Referral at the PHC level

Health workers in PHC make the decision about treatment and recommendations, classifying children according to their national IMCI guidelines. According to these, all severe cases (respiratory or non-respiratory) diagnosed either clinically or if the SpO2 level is < 90% must be urgently referred to hospital, and non-severe cases (classified as yellow and green) will have an oral prescription for treatment and guidance for home care status. Parents or caregivers of yellow cases will be advised to return to the PHC if there is no improvement in clinical signs in the three to five following days according to countries IMCI Guidelines.

All AIRE health facilities (PHC and DH) will be supported with the appropriate treatment (e.g. injectable antibiotics, oxygen therapy), but the intervention will be carried out under field care conditions, according to the national directives in force, and without substituting the national health system. This means that referral to hospital will not be directly managed by the AIRE project, but existing solutions will be promoted locally. This will allow to guaranty sustainability of the results in scaling-up the PO deployment.

- Step 4: At District hospitals

If the child is referred to the district hospital, the health care provider will administer according to needs, oxygen therapy, antibiotics, and the medications required to treat any other comorbidities to the newborn or child, and in case of hypoxemia will monitor the SpO2 at entry and during hospitalization.

- Step 5: Return to home

The health care provider will discharge the newborn or child at home once he/she is stabilized, with a counter-transfer back to their initial PHC.

### Objectives of the AIRE research protocol

The main objective of the AIRE operational research protocol is to evaluate the process of systematic use of PO (with hospital referral if hypoxemia is diagnosed with SpO2 < 90%, or if children is classified as a red case using the clinical IMCI classification based on paper or electronic support) during the IMCI consultation in children under 5 years old at primary health centers (PHC) in four West African countries. This research protocol will include three complementary components: a) evaluation of the context (infrastructure, personnel, use of PO, characteristics of IMCI’s outpatients), b) process evaluation (acceptability, fidelity, implementation challenges, realist evaluation) and c) outcome evaluation (care pathways, patterns of care, costs of care, and day-14 health outcomes for severe cases). These three components are based on the Donabedian model (context, process, outcome) often used to assess the quality of care [37].

Overall, the AIRE project will provide research evidence, using mixed quantitative and qualitative methods to answer the following questions measured in field conditions of PO use at the PHC level: why and how routine PO use into IMCI works (or not), for whom, in what contexts and with what results? In each component, we will specifically explore the differences when using e-IMCI vs paper-based IMCI. This will allow a better understanding of the strengths, weaknesses, opportunities and threats of the PO intervention as a whole, and the action levers for an optimized scaling up of the PO implementation at the country level.

#### Specific objectives of the evaluation of the PO implementation

- The assessment of the context aims to describe at baseline and over the study period all the AIRE sites introducing PO: health infrastructures, nursing staff, frequency of use of PO and characteristics of IMCI outpatients.
- Process evaluation aims to:
  Assess the level of acceptability of the innovation (PO) introduced by the project among health workers and caregivers.
  Describe the fidelity of the implementation, i.e. to what extent the final implementation is in line with the planned activities of the AIRE project (check whether the activities planned by the project have actually been implemented, and also often and for as long as originally planned).
  Analyze how the implementation of the AIRE project is organized, what are its challenges, what are the factors that influence it (including e-IMCI vs paper-IMCI).
  Understand how the AIRE project works, for whom, in which specific contexts and why, using a realist evaluation.
- The outcomes evaluation in research sites aims to:
  Describe the characteristics of eligible children for PO use during the IMCI consultation, their classification using IMCI alone, then using IMCI and PO, comparing their effect on hospital referral decision.
  Evaluate the care pathways from the first symptoms, and their correlates for IMCI outpatients and for all severe cases of any etiology (non-respiratory and respiratory) identified at the PHC level. This will include the added value of using PO for e-IMCI guidelines versus paper-IMCI guidelines.
  Evaluate the patterns of care (use of PO, oxygen therapy, medications, exams) and their associated factors for both IMCI outpatients and for all severe cases of any etiology (non-respiratory and respiratory) identified at the PHC level.
  Assess the frequency and correlates of successful transfer to hospital of severe cases and the reasons for missed opportunities.
  Measure the direct costs (medical and non-medical, including hospital transfer for severe cases) and indirect costs from a societal and health system perspective for managed cases (severe and non-severe), including the deployment of PO.
  Assess the health outcome on day-14 after the index consultation (death, alive still under care, alive cured, lost to follow-up) and correlates for all severe cases.

## Methods of the AIRE research protocol

### The AIRE study sites

**Fig. 3:**
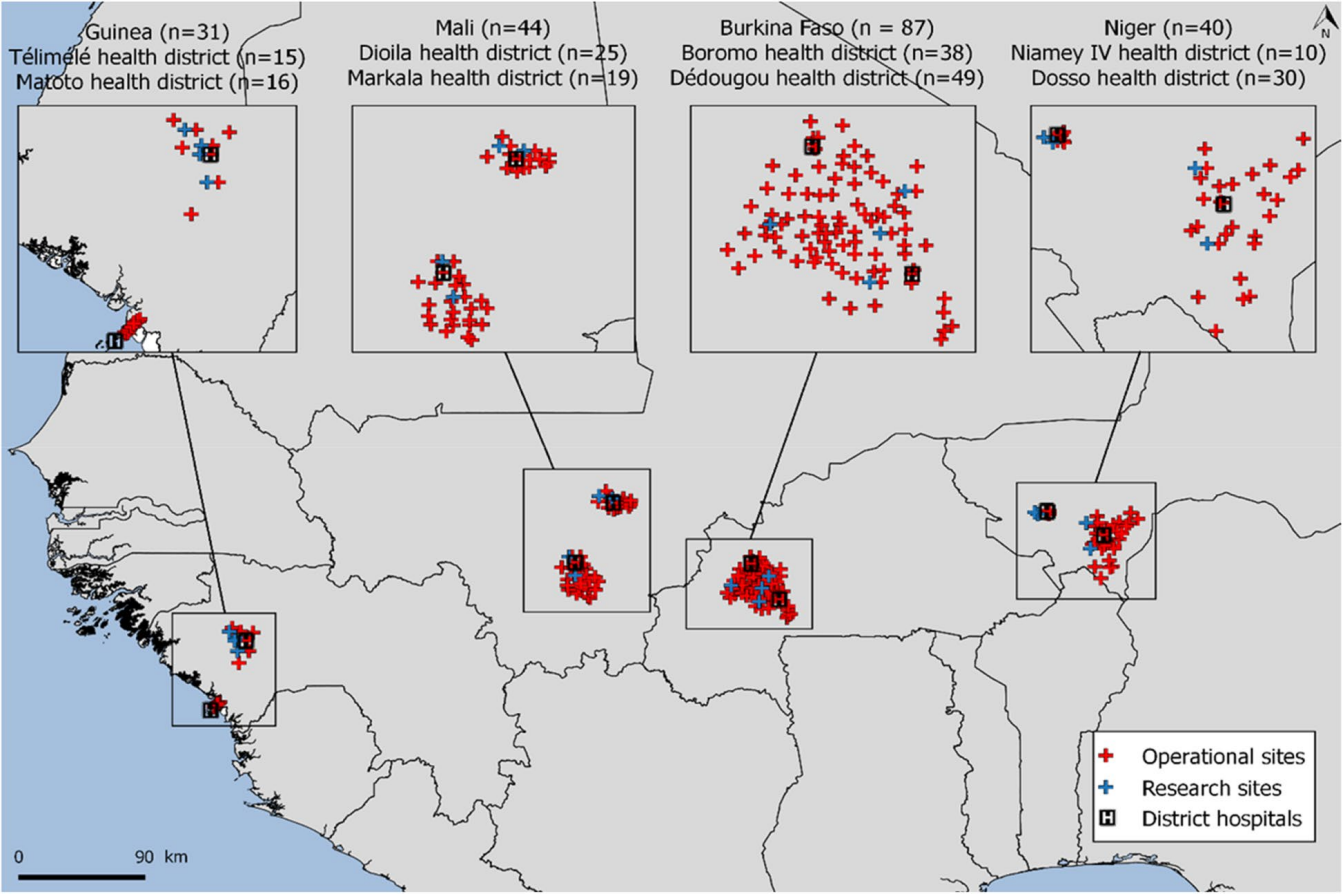
Map of intervention sites in the four countries participating in the AIRE project. Burkina Faso, Guinea, Mali, Niger, March 2021–December 2022. Source: Flore-Apolline Roy who gave her written permission to use it

The AIRE operational research study will be conducted in four AIRE countries where there is a high severe disease incidence: Burkina Faso, Guinea, Mali, Niger. The project will take place in two health districts per country, in a total of 8 district hospitals and 202 PHC (Fig. 3). All PHC will be considered as operational sites (from which aggregate health data will be collected) and 16 research sites will be selected among them (4 sites per country providing also individual health data). Data on the quantitative acceptability of HCW will be collected in both operational and research sites.

All project PHC have been selected if they were offering IMCI consultations to children under five and with the agreement of the national authorities. Among these, the research sites were selected according to the following criteria: i) public PHC, ii) PHC fully equipped with the equipment required for the implementation of IMCI consultations, iii) PHC in an area with Internet network coverage available for electronic data collection, iv) PHC accessible to the research team in terms of safety, geographically and for other reasons, regardless of the population accessibility, v) PHC without prior introduction of PO, vi) PHC with no other ongoing research project, vi) PHC with minimal hospital referral management capacity. To ensure size representativeness compared to other PHC at each health district level, a large PHC and a medium/low frequency PHC were selected as research sites. Thus, 16 research sites were selected by the AIRE research team based on the above criteria based on the AIRE site assessment. All research sites were also approved by the national authorities. In Guinea, the AIRE project also implemented specific COVID-19 activities through 29 PHC, including a PO implementation component, but introduced at the triage stage and using a SpO2 threshold of 94% for hospital referral, which differs from the WHO IMCI guidelines. Consequently, these latter sites were not included in the AIRE research sites. A description of AIRE intervention sites per country is summarized in Table 1.

**Table 1:** Description of AIRE intervention sites, primary health care centers (PHC) and district hospitals according to countries; March 2021–December 2022

Country	Burkina Faso		Guinea		Mali		Niger		Total
Health districts	Dedougou	Boromo	Matoto	Telimele	Dioila	Markala	Niamey IV	Dosso	
# of operational PHC	49	38	16	15	25	19	10	30	202
# of research PHC	2	2	0	4	2	2	2	2	16
# of district hospital	1	1	1	1	1	1	1	1	8
Type of IMCI support	electronic		paper		paper	electronic	paper		–
NGO implementer	Terre des hommes		ALIMA		ALIMA	Terre des hommes	Solthis		–

### The AIRE study design

The design of the AIRE operational research study will incorporate data from several cross-sectional studies and one prospective interventional cohort study with short-term 14-day follow-up for the severe cases. The recruitment and collection of mixed quantitative and qualitative data will be carried out over a period of 22 months from March 2021 to November 2022 in the four countries. The collection of individual IMCI data on children carried out over a period of 12 months (June 2021 to June 2022) will allow to consider any effect of seasonality for pneumonia, malaria and malnutrition.

#### Context evaluation

First, an exhaustive cross-sectional baseline survey of infrastructures, health personnel and site organization conducted in all PHC and referral hospitals where the AIRE project will be implemented (AIRE site assessment) was carried out before the introduction of the PO between March and July 2020. The objective was to map the project sites, to guide the selection of research sites and to assess the needs for upgrading the centers.

Secondarily, a prospective description of monthly PHC-level indicators will be provided for all AIRE sites after the introduction of the PO using aggregated data over the entire period of children’s enrollment: infrastructure, equipment (PO and oxygen availability), staff changes, and all IMCI consultation features.

#### Process evaluation

The AIRE project is a complex intervention, which involves a large number of actors, aims to implement plenty of activities and can be influenced by many elements external to the project (national policies, other Non-Governmental Organization (NGO)’ interventions, socio-economic context, etc.). To deal with this complexity, process evaluation will use mixed methods and will be based on a theoretical approach to evaluation [38] and more particularly on a realist approach to evaluation [39]. This approach aims to understand why an intervention works (or not), how, for whom and in which contexts.

A realistic evaluation relies on the principles of scientific realism [40]. It is based on the idea that an intervention does not work by itself and is not what produces an effect. An effect is the product of the interaction between a mechanism triggered by the intervention and the context in which the intervention is implemented. A mechanism must be understood as the way in which the actors involved in an intervention reason and react with regard to the resources available within the framework of this intervention [41]. It therefore aims to observe, in a particular context (C), the regular appearance - although not systematic - of a result (O), by triggering a mechanism (M). This view of causality is called generative. The objective is to develop Context-Mechanism-Outcome (CMO) configurations, to explain the intervention and its results. In fact, the realist research will start with a description on the intervention theory of the AIRE project and will lead to the production of a mid-range theory. Intervention theory is “the set of beliefs and assumptions that underpin program activities. Programs are inevitably based on a theory [...] of how activities are supposed to bring about the desired changes” [42].. Mid-range theory, proposed by Merton [43], can be defined as “the level of theoretical abstraction that provides an explanation of observed patterns and regularities in the context-mechanism-outcome interactions of a set of interventions “ [44].

The evaluation of the process will be based on a review of the theoretical and conceptual literature in order to formulate theoretical propositions that will guide the collection of empirical data. For example, we will draw on realist assessments and balances of oxygen delivery to children to develop theoretical propositions specific to the AIRE project [24, 27]. We will also build on the work of a realist evaluation of the IeDA approach to the management of childhood illnesses in Burkina Faso [45]. We will then empirically verify the validity of the various frameworks and theories by confronting them with empirical data using an abductive approach (back and forth between empirical data and theory to produce an explanation of a phenomenon). During these stages, we will involve the country and coordination teams through a participatory approach.

We have chosen to investigate more specifically several lines of research that will then allow us to feed the realist evaluation of the project: acceptability, fidelity of implementation and process.

#### Outcomes evaluation

A population-based cross-sectional study of all children under 5 years of age managed will be conducted in the 16 research PHC implementing the integration of PO into IMCI (electronic or paper) to assess: pathways, patterns, costs of care. A prospective longitudinal follow-up of severe cases until Day-14 after the index IMCI-consultation will document their health outcomes.

##### Study population and patient inclusion process at research sites

**Fig. 4:**
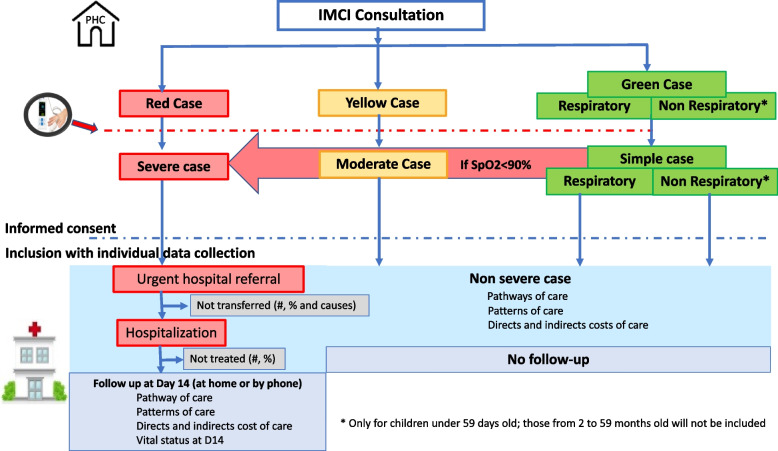
Inclusion process of children and follow-up in the AIRE research sites. Burkina Faso, Guinea, Mali, Niger, June 2021–June 2022

Screening and inclusion criteria process (Fig. 4)

All the neonates (0 to 59 days) and children (2 to 59 months) attending IMCI consultation in the research sites of the AIRE project will be screened by the HCW in place at the PHC. They will be classified as “red case”, “yellow case” or “green case”, according to routine IMCI guidelines based on their age category.

PO will be introduced after IMCI classification for all children based on age and danger signs, except for those aged 2–59 months and classified as non-respiratory “green case”. Children could be then reclassified as severe cases using PO if SpO2 < 90% defining hypoxemia, as shown in Fig. 4 (red arrow).

Eligible children for whom at least one caregiver gives signed informed consent will be enrolled consecutively during the recruitment period, from 2021, 14th June to 2022, 20th June.

All individuals identified as severe cases (respiratory and non-respiratory) under IMCI guidelines only or using PO should be referred to the hospital: these children will be included in a short-term follow-up study until Day-14 after the index IMCI consultation, regardless of their subsequent hospitalization or not, to document their health outcomes. Data on referral to hospital, and hospitalization of the child, if applicable, will be collected. In the event of hospitalization, care for children with serious respiratory or non-respiratory diseases (oxygen therapy and antibiotic therapy, etc.) will be provided under local conditions with the support of the AIRE project. At Day-14, study team will call or visit the caregivers at home to collect data on the child health status and care pathway through Day-14. Non-severe cases identified at the PHC will return home after the IMCI consultation without further follow-up.

Follow-up supervision, training and data quality control will be provided by the clinical supervisor at operational sites, and clinical research monitors and research assistants at research sites.

### Data collection, management and procedures

#### Context evaluation

A baseline descriptive study using a quantitative e-questionnaire for all PHC and another e-questionnaire for district hospitals has been conducted. The objective of these questionnaires was to describe (non-exhaustive list): distance among PHC and the hospital district, electrical power, its functioning and its capacities in terms of health personnel, supply and delivery services, the type of IMCI used, the PO use, usage of specific public health policies (access to care and prices), capacities and methods of hospital transfer.

Then, all the sites will collect monthly routine aggregated health indicators over the inclusion study period, with a description of the infrastructures, personnel changes and all the characteristics: number of IMCI consultations, number of IMCI consultations using PO, number of respiratory and non-respiratory severe cases, and number of hospitalizations decided by the HCW and accepted by families, stratified by age groups and sex. In all AIRE project sites (operational sites and research sites), this aggregated health data will be extracted from routine activities collected under the responsibility of Ministry of Health staff either at the PHC level or at the health district level.

The Inserm team will develop an operational monitoring tool for sites with REDCap® to be used in each participating site by the country Monitoring & Evaluation officers and district teams to monitor monthly trends.

#### Process evaluation

##### Acceptability

We will first seek to measure and understand the acceptability (quantitatively at all sites and qualitatively in research sites) of the routine use of PO integrated into IMCI by health workers and people who accompany children to PHC. Indeed, the process of acceptability or non-acceptability of an innovation is crucial for understanding the adoption and dissemination (or not) of an innovation. For this study, we will use a conceptual framework built from a scoping review [46] and with the framework’s best-fit synthesis method [47]. We will study acceptability in quantitative terms (via questionnaire based on a Likert scale in all sites), and in qualitative terms (via in-depth interviews, only in research sites). We will analyze acceptability at three time-points: prospective (before PO introduction), concurrent (approximately 6 months after PO introduction), and retrospective (approximately 18 months after PO introduction, and 2 months after the end of the data collection related to child monitoring).

All HCW working in the AIRE sites will therefore be interviewed as part of these sequential surveys. Concerning the child’s accompanying person, a stratified sampling (the strata being linked to variables such as the distance between the place of residence and the PHC, the child’s stage of severity, etc.) will be carried out.

##### Implementation fidelity

Our study of the fidelity of the implementation of the AIRE PO intervention will be carried out in the 16 research sites. It will be based on the analysis framework of fidelity and adaptation to the implementation of Carroll et al. [48] and Perez et al. [49]. We will first list the activities considered essential to the project, based on the official documentation of the AIRE project (implementation guide, action plans, meeting minutes) and interviews with the teams. For each activity listed, we will specify the content (activity implemented), the coverage (population reached by the activity) and the temporality (timing and duration of implementation) in order to assess whether the “active components” of the intervention were received by the “beneficiaries” as often and as long as they should have been. Empirical data will be collected with an implementation fidelity grid and for each activity, respondents will be asked to provide a detailed and documented assessment of its implementation at three levels over the study period: implemented as planned, not implemented as planned or modified, and not implemented. They may also mention other activities that have been added.

##### Process and realist evaluation

The process evaluation will focus on the challenges of introducing and disseminating PO according to the work of Greenhalgh et al. [50–53]. The seven conceptual dimensions presented in their framework will serve as a guide to better understand these challenges, including their influence on the adoption (or not), abandonment, diffusion, scale-up and sustainability of using PO during IMCI consultations.

The realist evaluation will be undertaken based on this same data collection, but will be conducted in accordance with the intervention theory and design approach presented above. For this dual analysis, qualitative empirical data will be collected in the 16 research sites. Data will be collected through interviews and field observations at the 16 research sites to understand how the PO intervention is actually implemented and used in these PHC.

#### Outcomes evaluation

##### Individual data collection

Once enrolled in the AIRE research study, the following data will be collected for each child by the data collector from Day-0 at the PHC level (Table 2): baseline socio-demographic and clinical history data, specific IMCI signs and symptoms and care provided according to the age-specific IMCI classification of national guidelines and WHO guidelines [29, 30, 34, 35]. Non-severe cases identified at the PHC will return home after the IMCI consultation. Then, all severe cases (respiratory and non-respiratory) will be followed up until Day-14 after the index IMCI consultation to document: hospital transfer modalities; pathways of care; models of care at PHC and hospital level; and short-term health outcomes in terms of vital status at Day-14 (Table 2). In all research sites, quantitative and qualitative data will be collected through electronic Case Report From (e-CRF) by specific research data collectors based at PHC level, and a counsellor to ensure the Day-14 vital status follow-up either by phone, or home visit if needed. Each health district has at least one referral health center or district hospital (DH) for the referral of severe cases to be treated according to the adapted WHO guidelines per country [4, 54]. Data will be collected on all patients referred from the PHC research sites to the DH by a research data collector based at each DH.

**Table 2:** Collection of individual data according to the children schedule at the AIRE research sites

	Inclusion All IMCI eligible consultation at PHC level	If hospitalized, recommended for all severe cases (respiratory and non-respiratory cases)		Follow-up visit: at the PHC, telephone call or home visit All severe cases regardless of hospitalization
	D-0	Entry	Discharge	Day-14 (+ 14 days)
Inclusion criteria Informed consent	✓			
Socio-demographic characteristics, care pathway before attending PHC, IMCI classification	✓			
Medical history and treatment	✓			
Clinical examination (symptoms and signs) ^a^	✓	✓	✓	
Co-morbidities (malnutrition, anemia, malaria)	✓	✓		
SpO2 Measurement by PO and integration into the IMCI classification (severe case if SpO2 < 90%)	✓	✓	✓	
Use of Malaria Rapid Diagnostic Test (RDT)	✓	✓		
Medical treatment (antibiotics, artemisinin -based combination treatment, salbutamol, etc.)	✓	✓	✓	
Hospital referral decision for severe cases	✓			
Missed opportunities of referral for severe cases and reasons				✓
Oxygen therapy		✓	✓	
Laboratory evaluation, (full blood count, malaria RDT…) if applicable		✓		
Evaluation of response to treatment		✓	✓	
Treatment and management of comorbidities		✓	✓	
Costs (households, and government perspective) ^b^	✓	✓	✓	✓
Vital status			✓	✓

^a^ content of the clinical assessment changes depending on the place of examination

^b^ representative sub-sample of non-severe and severe cases

In addition, a cost study will be conducted for a sub-sample of severe and non-severe cases in research settings from a societal perspective, but also the cost paid by governments and the costs borne by households. Direct medical costs (medical consultations, medicines, hospital transfer, hospital stay), direct non-medical costs (transport to health centers and hospitals, accommodation) and indirect costs (loss of income) incurred by households will be collected. Direct medical costs will include those incurred by the health system (infrastructure equipment, examination and care, PO use, staff costs). From a societal perspective, cost will include the intervention costs (cost of setting up PO in health centers, staff training, equipment, maintenance of equipment, community intervention), direct medical and non-medical costs as well as indirect costs [55]. The cost of soliciting caregivers and the consequent loss of income will be collected through interviews. Expenditure incurred by households will be estimated declaratively using a standardized questionnaire. Activity mapping and resource use will be assessed through semi-structured interviews and guided focus groups. Qualitative information will be collected until saturation is reached. All costs will be expressed in 2021 international dollars. Quantitative cost data will be collected by data collectors under the supervision of health economists. Health economists will estimate the additional project cost intervention through deliberate sampling.

All data collected will be anonymized and stored in the REDCap® database.

##### End of search

All enrolled children, classified as severe cases will be followed up to 14 days after inclusion. The official end of the research, except in the case of premature termination, is defined as the last day of follow-up Day-14 of the last child included in the study.

##### Withdrawal from the study

An investigator may remove a subject from some or all study components at any time at their discretion. In such a case, the investigator will inform the parent(s)/guardian(s) that the child has been withdrawn from participation in the study, and the reasons therefore. In any case, the clinical management of the child will be prioritized over research.

The parent(s)/guardian(s) can decide at any time to withdraw the child from the study if they wish, without justification nor any consequence on the quality of follow-up and subsequent care. In this case, no new information should be collected and recorded in the database after the date of withdrawal. They could also expressly request to delete all previously collected data.

##### Loss to follow-up

A child not withdrawn by consent, deceased or transferred, and whose family does not show up or respond for the follow-up on Day-14 (+ 14 days) will be considered as definitely lost to follow. The follow-up date will be the date of their last contact with the study team (either at the hospital, by phone or at home).

### Sample size

#### Context evaluation

All AIRE PHC in two health districts per country, ie 202 PHC and 8 district hospitals, and their staff concerned were evaluated at baseline and will then be evaluated monthly until the end of the project.

#### Process evaluation

- Quantitative acceptability (prospective, concurrent and retrospective): all HCW in charge of using PO in the 202 PHC.
- Qualitative acceptability in the 16 research PHC (concurrent and retrospective): approximately 64 HCW (approximately 4 per research site) and approximately 64 caregivers (stratified sample to respect the diversity of their situation: age, sex, distance/familiarity with the PHC, health status of the child…).
- Fidelity of implementation: all HCW in the 16 research PHC, people working with the PHC (managers of drug depots, management committee managers, etc.) and people working at the health district level
- Process and realist evaluation: approximately 20 people per district (HCW, caregivers, members of the management committee, community health workers, etc.).

The principle of empirical saturation (learning nothing new about the subject), usually used in qualitative study will be applied.

#### Outcomes evaluation

Cross-sectional study of pathways and patterns of care will be conducted among all IMCI children eligible for PO use with caregiver consent by research PHC. Children will be recruited consecutively during the 12-month.

Direct and indirect costs will be collected for a representative sub-sample of two categories of children: IMCI outpatients (green and yellow cases) and severe cases requiring hospital transfer (5 per category per PHC per month, i.e. 1920 children in total).

Day-14 health outcome will be collected only for severe cases. To assess the child mortality on Day-14 of all severe cases identified with an accuracy of plus or minus 3% per country and adding 10% lost to follow-up, we aimed to recruit at least 710 severe cases aged 2 to 23 months (mortality estimated at 10%) and 375 children aged between 24 and 59 months (mortality estimated at 5%), i.e. 1085 children between 2 and 59 months old per country over 12 months, for a total of 4340 children. We will only describe mortality in neonates (aged less than 59 days) enrolling them all over the 12 months of the operational study with a maximum of 400 per country. Overall, we will follow a maximum of 1485 children per country, i.e. a maximum of 5940 severe cases over the study period, representing approximately 15% of the total IMCI population which will be a total of 40,000 children expected for the four countries.

### Data analysis

#### Context evaluation

We will carry out a baseline description of the infrastructure of the PHC: number and % of PHC having access to electricity or the Internet, average distance between the PHC and the DH, a map of all the health facilities). Furthermore, we will conduct a description of the functioning of PHC and HCW: number and % of PHC using electronic compared to paper-based IMCI, hospital transfer capacities and modalities, health services, qualification of HCW and average number of HCW per PHC.

We will describe the functioning of the PHC and staff turnover over the study period: in % compared to baseline. The epidemiological characteristics of IMCI consultations will be described stratified by country and disaggregated by age (0–59 days, 2–59 months) and sex (male, female), mean monthly number of IMCI consultations and standard deviation, with % of PO uptake, mean monthly number and prevalence of severe cases globally and according to the type of IMCI (paper or electronic), and after the use of PO, their type (respiratory or not) identified with indication of hospital referral, coverage in % of decided hospital transfer among the severe cases identified.

#### Process evaluation

##### Acceptability

Quantitative acceptability data will be analyzed using descriptive and inferential statistics. Regression models will help understand the individual and contextual determinants of acceptability. An analysis of the difference in acceptability before and after the implementation of the PO as well as after the end of the project will make it possible to measure the effects of the implementation of the project on the prospective, concurrent and retrospective acceptability. Qualitative data will be analyzed using an analytical framework analysis approach [56] to compare the empirical data with the theoretical model used for data collection through Nvivo software.

##### Implementation fidelity

The data will be analyzed according to the method of the analytical framework [56] using the framework adapted from Carroll et al. [48]. Qualitative data on the fidelity of activity content will be converted into quantitative data by considering the proportions of activities ranked by respondents as 1) implemented as planned; 2) not implemented; 3) planned but modified; and 4) added. A fifth option, “non-response” will be added to account for missing responses. Temporal fidelity will be examined in terms of frequency (eg monthly, quarterly) and if possible duration, understood as the number of days planned for this activity. The results will be presented graphically for each district and country.

##### Process and realist evaluation

The qualitative data will be fully transcribed and then integrated into the NVivo software to analyze the data and compare them with the theoretical model of Greenhalgh et al. [52]. For the realist evaluation and development of the final Mid-Range-Theory, an abductive approach will be used to identify the Context – Mechanisms – Outcome configurations from the existing theories in the literature and from all the data collected in the project, in order to highlight the contexts in which the mechanisms are (or are not) triggered, and the resulting outcomes. This approach aims to construct a theoretical explanation of a phenomenon. This dialogue between theoretical literature and empirical data will allow us to build and gradually refine the Mid-Range-Theory. The semi-regularities will be identified in the data collected by the content analysis of the NVivo software and then modeled in a process-oriented way. More specifically, we will take up the four steps proposed by Abimbola et al. [57] to: 1) identify the effects; 2) identify the contextual components related to the effects; 3) theoretically reconstruct the links between the context and the effects; 4) identify the mechanisms.

#### Outcome evaluation

##### Descriptive analysis

The analysis will first describe and analyze each of the following results stratified by country:

- Characteristics of children under 5, eligible for PO during IMCI consultation: distribution by age, sex, distance from home to PHC, socio-demographic variables, rural/urban PHC, clinic IMCI classification, type of case (respiratory or not), PO uptake (%), SpO2 level measurement (mean and % of hypoxemia [severe< 90% and moderate 90 to 93%]) and IMCI+PO classification, IMCI management (on paper/electronic), severe cases (respiratory and non-respiratory) and rate among severe cases with hospital transfer indicated.
- Pathways of care (in %) stratified according to severity (IMCI+PO) and hypoxemia: history of recent attendance at PHC, duration since the onset of symptoms, effective rate of hospitalization among severe cases (proportion of hospitalizations carried out among all those identified at PHC with a hospital transfer indication, missed referral opportunities among severe cases with indication for hospital referral and reasons for non-transfer, return to PHC care, or rehospitalizations.
- Patterns of care for IMCI outpatients stratified according to severity (IMCI+PO) and hypoxemia, and for inpatients, hospitalization patterns: coverage of oxygen therapy, antibiotics, ACTs, and other specific treatments in %, volumes, mean time between the medical treatment and onset of symptoms, and the diagnosis of hypoxemia and mean durations of resources utilization.
- Costs expressed as medians with interquartile ranges, according to the severity of the case, hypoxemia, and the hospitalization by country
- Short-term health outcome (deceased, alive and still treated, alive and cured, lost to follow-up) on Day-14 (in %) for all severe cases identified at the PHC level.

Descriptive statistics will include proportions for categories (with their 95% confidence intervals) and means (standard deviation) or medians (interquartile range [IQR]) for continuous variables. Differences in proportions will be tested by the Chi-square test (or Fisher’s exact test in the case of small sample sizes), and continuous variables will be compared using the t-test or Wilcoxon rank-sum test, as applicable.

##### Analysis of associated factors to the main criteria

- Factors associated with being a severe case (using paper or e-IMCI and using PO or not) among IMCI outpatients, being hospitalized among severe cases, non-referral among severe cases will be studied using a multivariate logistic regression, adjusting for clustered variables (PHC, support of IMCI paper or electronic, rural/urban location), and for individual data (age, sex, socio-demographics, IMCI classification illness, comorbidities, seasonality, distance from home to PHC or district hospital, SpO2 level threshold using PO,...), using robust standard error to take into account the cluster effect within each PHC. All *P* values will be two-sided, with P values < 0.05 considered statistically significant.
- Kaplan-Meier estimates of the cumulative probability of oxygen therapy delivery and Day-14 survival will be performed for all identified severe cases with hypoxemia, whether hospitalized or not. Day-14 mortality associated factors will be analyzed using logistic regression. Additionally, in a sensitivity analysis missing data will be handled by using a complete data analysis and will consider the maximum bias hypothesis replacing missing data or dropouts by death values.
- The pathways (hospital transfer) and patterns of care (access to oxygen therapy, volume, duration) will be analyzed and their correlates investigated using a logistic regression.

##### Cost analysis will describe and analyze

- The median overall direct cost of care per child managed (outpatients visits, hospitalization days, drugs, and oxygen, others, …) will be estimated (in USD) in each country according to the different categories of patients (non-severe cases, severe cases not transferred, and severe cases hospitalized). Within each category, we will investigate the effect of the support of IMCI used (paper or electronic) and PO use leading to IMCI reclassification. Indirect costs will be similarly estimated for the severe and non-severe cases.
- We will compare costs for managing non-severe cases, severe cases not transferred, and severe cases hospitalized using a mixed linear regression model.
- The PO implementation cost will be assessed exploring the additional cost of PO use for the health system and the remaining costs for households according to the severity of cases, the pathway of care and the patterns of care and will be studied in the 4 countries of the project.

### Ethics and consent to participate

The AIRE research protocol, the information notice (translated in vernacular languages), the written consent form and any other relevant document have been submitted to each national ethics committee, to the Inserm Institutional Evaluation Ethics Committee (IEEC) and to the WHO Ethics Review Committee (WHO-ERC). All the aforementioned ethical committees reviewed and approved the protocol and other key documents (Comité d’Ethique pour la Recherche en Santé (CERS), Burkina Faso n°2020–4-070; Comité National d’Ethique pour la Recherche en Santé (CNERS), Guinea n°169/CNERS/21; Comité National d’Éthique pour la Santé et les Sciences de la vie (CNESS), Mali n°127/MSDS-CNESS; Comité National d’Ethique pour la Recherche en Santé (CNERS) Niger n°67/2020/CNERS; Inserm IEEC n°20–720; WHO-ERC n° ERC.0003364). all ethical approval and NICE were attached in the Study protocol proof section in the submission system. This study has been retrospectively registered by the Pan African Clinical Trials Registry on June 15th 2022 under the following Trial registration number: PACTR202206525204526.

All individual interviews will be conducted after explaining the relevance of the research and asking for the free and written informed consent of the interviewees. The original consent form will be kept by the site’ Principal Investigator (PI) and NGO in a secure location inaccessible to others, even when moving, throughout the study period and up to 15 years after the end of the study.

Parents or guardians shall be informed of their right, during or after the research, to receive information concerning the health of their child held by the investigator or, where applicable, by the qualified person representing the investigator. If the child is in an emergency situation, the consent process will not be conducted until the child is stabilized. In all cases, the clinical management of the child will take priority over the research. Any clinically significant anomaly detected in the results of the examination or test will be communicated to the parents/guardians and the doctor chosen by them, unless they object.

Participants will not receive any financial reward for their participation.

All data will remain confidential and will only be accessible to researchers via secure access. All agents involved in the collection and/or management of data will be required to sign a confidentiality agreement. The data will be anonymized after collection and kept for 15 years following the end of the study. A copy of the data will be stored on a virtual platform and protected by a password.

## Discussion

AIRE research protocol will provide useful and original information on the introduction of routine PO use into IMCI consultations at PHC level by addressing several West African specificities compared to other regions of sub-Saharan Africa:

- Children mortality rates in West Africa are among the highest in sub-Saharan Africa and strongly correlated with indicators of poverty, underlying malnutrition and infectious diseases [58–60]. The disease burden is complex. Fever-related illnesses remain one of the leading causes of death in children under 5, including malaria and pneumonia. Malaria is particularly acute in West Africa [61] with prevalence ranging between 30 and 64%. Sickle cell disease is also widespread and is an invisible and underestimated cause of anemia and a neglected cause of morbidity in children under-5 in West Africa [62, 63]. This adds to the context of malnutrition often coupled with specific nutritional deficiencies (lack of iron [64], folates and other micro-nutrients) and the presence of other parasites (especially helminths) playing an underestimated role in “chronic epidemic anemia” [65], which is an underlying factor associated with almost half of deaths of children under-5 in West Africa. Second, all these comorbidities including hypoxemia are frequently neither identified nor treated contributing synergistically to the fatal worsening of severe cases among children in West Africa.
- The West African health system is also weak with insufficient health resources, low access to health services and insufficient investment in frontline health care. There is a critical shortage of health workers, particularly at the primary health care level and in poor rural areas. As training programs progress, qualified personnel often leave to work elsewhere [66]. This leads to issues of health equity, where there are currently many underserved children and often inadequate quality of care [66, 67]. Inadequate clinical capacity (i.e. inadequate implementation of IMCI guidelines) to identify severe cases in neonates and children means that children with danger signs or signs not specific and with severe illness (including malaria, pneumonia, and other infectious diseases) are missed or misdiagnosed at PHC level and not referred to hospital [67]. In the context of the COVID-19 pandemic, the West African health system has been further disrupted, with an expected indirect impact on both delivery and demand for routine IMCI care among children under-5 that needs to be better described and understood [68].
- Finally, the cost of care could impact access to care in West Africa: health care for children under-5 is free for families in Niger and Burkina Faso but not in Mali and Guinea except for malaria, tuberculosis, HIV and malnutrition. However, the user fee abolition policy is currently facing a major obstacle such as the sustainability of financing which impacted the cost of care [69]. As it is standard practice to ask families to pay for care at the point of service, the capacity to pay is a major determinant of access to care, particularly for hospital referral creating major social inequities [70]. Generally, urban versus rural populations, and those with higher socio-economic status (SES) and education are more likely to seek care with health providers than lower SES groups or illiterate population. For example, the 2012 Demographic Health Survey in Mali reported that among children who experienced fever in the 2 weeks preceding the survey, 43% of urban populations sought care from a health provider, versus only 29% in rural areas [71].

The initial AIRE research protocol submitted (V1.3 - 5th March 2020, approved by the four National Ethics Committees) before the COVID-19 pandemic, was built on a quasi-experimental comparative before-after PO introduction study design (with three research components assessing the process, impact, and cost-effectiveness). Unfortunately, because of the COVID-19 pandemic having emerged in March 2020, this protocol was biased and the project delayed. To mitigate this bias, we therefore proposed a revised operational non-comparative research study protocol that was consequently also approved and launched in 2021.

However, several issues were discussed during the AIRE study design development that will not be fully addressed:

1. When is the best time to introduce the PO into the IMCI process?

The initial ideas were to introduce PO measurement at the entrance to the IMCI consultation for operational ease, rather than afterwards. The reasons for using PO after examination and classification of the child have been suggested by the International Advisory Group (IAG) as follows: a) the risk of having false positive hypoxemia (1 to 5% of the measurements according to an unpublished data) and therefore sending the child in the hospital without real need, and leading to a risk of overloading the health system if too many children are sent to the hospital, b) other adverse effects potentials for “overuse” of PO, mainly transmission of dirty hands disease between children if PO is not well disinfected; c) the risk that health workers will use SpO2 measurements to classify children without examining them; (d) the risk of overuse of the PO and of having to replace faulty devices more frequently. Thus, the introduction of the PO was systematically integrated after the IMCI consultation in our approved protocol without a comparison group at the time of entry. But the interviews of the HCW will better explore the ideal time to use the PO.

2. What SpO2 threshold should be used for hospital referral?

The choice of SpO2 threshold is critical but not obvious, as clinical signs may be difficult to identify at PHC level and may not be reliable predictors of hypoxemia. The 2014 WHO IMCI guidelines recommend providing oxygen therapy when SpO2 is less than 90%. However, this threshold will likely result in misclassification of some children, as cases with non-severe pneumonia when SpO2 ≥ 90% and who have undiagnosed comorbidities will be at high risk of death [72]. Although the quality of the evidence is very low, the WHO, based on hospital evidence [54], strongly recommended in 2016 that children with other emergency signs, with or without respiratory distress, receive oxygen therapy if their SpO2 is < 94%. At the PHC level, the field effectiveness of the SpO2 threshold is unknown; oxygen therapy is not available, and it is probably necessary to choose a more sensitive threshold to anticipate the delay before any actual transfer to the hospital where the oxygen will be delivered. In terms of implementation considerations, the recommendation to give oxygen to all children with emergency signs if SpO2 < 94% must also be weighed against the increased demand that would be placed on resources in the LMIC where oxygen supplies may be scarce [24]. Answering such a question would have required a comparative parallel study design comparing two different SpO2 thresholds strategies (90 and 93%). This study design was unfortunately not retained for ethical and logistical reasons. Additionally, since we will not be tracking moderate IMCI cases in the AIRE protocol, we will also not be able to assess their risk of misclassification.

3. How to deal with the situation of care and hospital referral?

The AIRE project will ensure that essential drugs are effectively provided and operational from the start of the project under the country ministries of health auspices: PHC will be equipped with medical equipment for IMCI consultations, antibiotics, ACTs and medical consumables to provide first aid before referral hospital. But increasing the detection of hypoxemia at the PHC level in West African settings raises the question of how to improve emergency oxygen availability or oxygen availability at the peripheral health level. Medical oxygen, which can save lives, is also in short supply in West Africa, especially in small hospitals with poor infrastructure and low human resource capacity. Access to hospital and the administration of oxygen to children who need it is an ethical duty and key elements when considering the implementation of PO at PHC level. This could have an impact both on the use of PO by HCW, and on the effectiveness on child health outcomes. As a result, pediatric unit and pediatric emergency rooms were equipped with injectable antibiotics, ACTs, other drugs, PO, and oxygen concentrators for the treatment of hypoxemia, aspirators and nebulizers at the hospital level. The management of hospital referral of severe cases from all PHC has raised problems in real life conditions: in fact, hospital referral brings a lot of social inequalities in access to health care because often at the family charge with high risk of catastrophic health expenditure in West Africa [73]. There is also an ethical duty to actually take charge of the severe cases that will be identified at the PHC level with the help of PO. This will also have a positive impact on the acceptability of health workers to systematically use PO beyond the end of the project. To address this dilemma, AIRE project suggested to identify actively and support existing local strategies from the start of the project, using and strengthening the local district health system to manage hospital referral.

In the end, the absence of a comparative group will not make it possible to assess the cost-effectiveness impact of using PO into IMCI management at PHC. In settings where resources dedicated to healthcare are limited, a cost-effectiveness analysis would provide a useful estimate of the efficiency of this intervention for decision-makers in West African countries. Furthermore, the selection of the PHC (geographically accessible, access to internet…) may have an impact on the generalization of the study’s results. These will be applicable to PHC, which are alike to those included in this study. In addition, the scaling up of the intervention will also require improving the technical capacity of PHC and district hospitals at the country level to achieve the expected impact of the intervention.

Despite these pitfalls, the current AIRE research project uses a comprehensive, context-specific public health research approach (context, process, outcomes) informing primarily about: process implementation, acceptability, pathways of care, patterns of care, short-term health outcomes, and costs of care, comparing type of IMCI (electronic versus paper versions). These data will provide sufficient evidence for national decision-makers to adopt operational changes that can and promote the timely and appropriate use of PO integrated into the IMCI strategy by HCW at the PHC level, and caregivers’ IMCI-seeking behaviors. The evidence generated is expected to result in scaling up beyond West Africa. This will include transition planning support and information; disseminate the results of the project at national and international level to integrate PO. In conclusion, we hope that the AIRE project will help to identify the brakes and levers of action to optimize the scaling up of an effective use of PO during the IMCI consultation at the PHC level in order to improve survival of children under-5 in the long term in West Africa.

## Supplementary Information

**Additional file 1.** The AIRE Research Group is composed as follows (as of 11/10/2022).

## Data Availability

Data sharing does not apply to this article as no datasets were generated or analyzed for the current study. The data will be made available by the corresponding author (Inserm) to any interested researcher upon reasonable request, and once the main outcome studies will be published. Anonymized participant data and a data dictionary would be made available and shared under a data transfer agreement.

## Abbreviations

AIRE: Amélioration de l’Identification des Détresses Respiratoires chez l’Enfant
ALIMA: Alliance for International Medical Action
ACT: Artemisinin-based combined therapy
CHW: Community healthcare worker
CMO: Context-Mechanism-Outcome
CERS: Comité d’Ethique pour la Recherche en Santé
CNERS: Comité National d’Ethique pour la Recherche en Santé
CNESS: Comité National d’Éthique pour la Santé et les Sciences de la vie
DH: District Hospital
e-CRF: electronic Case Report From
e-IMCI: electronic Integrated Management of Childhood Illness
HCW: Health care worker
IMCI: Integrated Management of Childhood Illness
IeDA: Integrated electronic Diagnosis Approach
IAG: International Advisory Group
IQR: Interquartile Range
Inserm: French National Institute of Health and Medical Research
Inserm EEC: Inserm Evaluation Ethics Committee
IRD: French National Research Institute for Sustainable Development
LMIC: Low- and middle-income countries
NGO: Non-Governmental Organization
PHC: Primary health center
PI: Principal Investigator
PO: Pulse Oxymeter
SaO2: Arterial Hemoglobin oxygen saturation
SES: Socio-economic status
SOLTHIS: Solidarité Thérapeutique et Initiatives pour la Santé
Sp02: Pulse Hemoglobin oxygen saturation
Tdh: Terre des hommes-Lausanne
WHO: World Health Organization
WHO-ERC: WHO Ethics Review Committee
