# Supplementary material for: Evaluation of the routine implementation of pulse oximeters into integrated management of childhood illness (IMCI) guidelines at primary health care level in West Africa: the AIRE mixed-methods research protocol
Source: BMC Health Serv Res. 2022 Dec 24;22:1579. doi: 10.1186/s12913-022-08982-4 (PMC9789366; doi:10.1186/s12913-022-08982-4)
Supplement: Supplementary file 1 — Additional file 1. The AIRE Research Group is composed as follows (as of 11/10/2022). [file 12913_2022_8982_MOESM1_ESM.docx]

**Additional file : The AIRE Research Group is composed as follows (as of 11/10/2022)**

**Country investigators**: Ouagadougou, Burkina Faso: Solange Yugbaré Ouédraogo (CHU de Bogodogo), Valérie Marcella Sanon Zombré (Direction Santé de la Famille, Ministère Santé), Conakry, Guinea: Mahmoud Sama Cherif (Centre de Recherche - Maferinya), Ibrahima Sorry Diallo (Institut National de la Santé de l’Enfant), Dieney Fadima Kaba, (Direction Santé de la Famille, Ministère Santé). Bamako, Mali: Abdoul Aziz Diakité (CHU-Gabriel Toure), Amadou Sidibé, (Direction Générale de la Santé, Ministère Santé). Niamey, Niger: Hannatou Abarry Souleymane (Direction de la Santé Néonatale et Infantile, Ministère Santé), Fati Tidjani Issagana Dikouma (Direction de la Santé de la Mère et de l’Enfant, Ministère Santé).

**Research coordinators & data centers: Inserm U1295, Toulouse 3 University, France:** Honorat Agbeci, Laura Catala, Désiré Lucien Dahourou, Sophie Desmonde, Emelyne Gres, Gildas Boris Hedible, Valériane Leroy (research coordination), Lucie Peters Bokol, Joséphine Tavarez, Zineb Zair. **CEPED, IRD, Paris, France:**Sarah Louart, Valéry Ridde (process coordination). **Inserm U1137, Paris, France**Anthony Cousien. **Inserm U1219,** EMR271 IRD, **Bordeaux University, France**: Renaud Becquet, Valérie Briand, Valérie Journot. **PACCI, CHU Treichville, Abidjan, Côte d’Ivoire** : Sévérin Lenaud, Célestin, N’Chot, Benjamin Seri, Cyrille Yao.

**Consortium NGOs partners:**

**Alima-HQ (consortium lead), Dakar, Sénégal**: Gildas Anago,  Dame Badiane, Moumouni Kinda, Désiré Neboua, Papa Semou Dia, Susan Shepherd, Naomi di Mauro, Grégory Noël, Karoline Nyoka, Woré Taokreo, Marine Vignon. **Alima, Conakry, Guinea:** Philippe Aba, Nanfandima Diallo, Masra Ngaradoum, Samuel Léno, Amadou Thierno Sow, Amadou Baldé, Aziz Soumah, Boubacar Baldé, Fatoumata Bah, Koumba Cécile Millimouno, Madeleine Haba, Mamadou Bah, Mariama Soumah, Marie Guilavogui, Mohamed Naby Sylla, Sadigatou Diallo, Sia Fatoumata Dounfangadouno, Thierno Ibrahim Bah, Sayadi Sani, Christian Gnongoue, Soda Gaye, Jean Paul Yassa Guilavogui, Amadou Oury Touré, Jacques Séraphin Kolié, Abdoul Salam Savadogo. **Alima, Bamako, Mali:** Freddy Sangala, Mahamane Traore, Tieting Konare, Adama Coulibaly, Aminata Keita, Djeneba Diarra, Hamed Traoré, Ibrahim Sangaré, Ibrahima Koné, Mariama Traoré, Souleymane Diarra, Victor Opoue, Fadiala Kalilou Keita, Melanton Dougabka, Bakary Dembélé, Mamadou Sériba Doumbia, Ganguinwiligba Désiré Kargougou, Sory Keita.

**Solthis-HQ, Paris**: Sandrine Bouille, Sophie Calmettes, Franck Lamontagne. **Solthis, Niamey:** Kadri Hama Harouna, Baraya Moutari, Idi Issaka, Sidi Ousmane Assoumane, Solange Dioiri, Sidi Mariama, Karima Sani Alio, Seyfourlaye Amina, Rolland Agbokou, Mahamadou Goli Hamidou, Salami Mamane Sani, Abdouramane Mahamane, Aboubacar Abdou, Barira Ousmane, Issa Kabirou, Issa Mahaman, Ibrahim Mamoudou, Moumey Baguido, Ramatou Abdoul, Amadou Sahabi, Fawziyah Seini, Zakaria Hamani, Lâang-Yerumianuo Bertrand Meda, Mactar Niome, Xavier Toviho, Issifi Sanouna, Patrick Kouam.

**Terre des hommes-HQ, Lausanne:**Sandrine Busière, Florian Triclin. **Terre des hommes, BF:** Adama Hema, Marc Bayala, Lucien Tapsoba, Jean Baptiste Yaro, Salifou Sougue, Richard Bakyono, Abdoul Guaniyi Sawadogo, Alimatou Soumah, Yayé Albert Lompo, Bibata Malgoubri, Florence Douamba, Gertrude Sore, Larissa Wangraoua, Sandrine Yamponi, Sarah Isabelle Bayala, Seydou Tiegna, Sie Kam, Solange Yoda, Mpafitini Karantao, Diane Fatimata Barry, Oumar Sanou, Noel Nacoulma, Nassirou Semde, Ibrahima Ouattara, Fidèle Wango, Zakaria Gneissien, Hassami Congo. **Terre des hommes, Mali:** Youssouf Diarra, Boubacar Ouattara, Alhamjiatou Maiga, Fatoumata Diabate, Ousmane Goita, Salimata Gana, Samba Diallo, Sidi Sylla, Dramane Coulibaly, Nadia Sakho.

**Country SHS team: Burkina Faso**: Kadidiatou Kadio, Josaphat Yougbaré, Daniel Zongo, Sylvain Tougouma, Aïchata Dicko, Zonabo Nanema, Ingrid Balima, Adidjata Ouedraogo, Abou Ouattara, S. Edmond Coulibaly. **Guinea**: Habibata Baldé, Lansana Barry, Etienne Duparc Haba. **Mali**: Abdourahmane Coulibaly, Tamba Sidibe, Yaya Sangare, Bintou Traore, Yacouba Diarra. **Niger**: Abdoua Elhadji Dagobi, Samira Salifou, Babba Gana Moustapha Chétima, Ibrahim Hadjara Abdou.
